# A laboratory-friendly protocol for freeze-drying sample preparation in ToF-SIMS single-cell imaging

**DOI:** 10.3389/fchem.2025.1523712

**Published:** 2025-03-07

**Authors:** Xiujuan Shi, Mingru Liu, Yue Qi, Hongzhe Ma, Zhaoying Wang, Yanhua Chen, Zeper Abliz

**Affiliations:** ^1^ Key Laboratory of Mass Spectrometry Imaging and Metabolomics (Minzu University of China), State Ethnic Affairs Commission, Center for Imaging and Systems Biology, College of Life and Environmental Sciences, Minzu University of China, Beijing, China; ^2^ Beijing Engineering Research Center of Food Environment and Public Health, Minzu University of China, Beijing, China

**Keywords:** ToF-SIMS, single cell imaging, sample preparation, freeze-drying, mass spectrometry imaging

## Abstract

ToF-SIMS is a high spatial resolution imaging technique for cellular or subcellular analysis of biological samples. Accurate molecular data in single-cell studies depend on proper cell morphology and chemical integrity, highlighting the importance of sample preparation. In this work, we standardized a more efficient freeze-drying method using standard lab materials and improved the sample preparation process. Our comprehensive freeze-drying protocol for cellular samples, encompassing washing, fixation, and drying steps, facilitates the acquisition of enhanced cellular information and ensures high reproducibility. These improvements are poised to significantly advance single-cell mass spectrometry imaging research.

## 1 Introduction

Single-cell imaging is increasingly becoming an important tool to help unlock biological complexity with the continuous advancement of single-cell research (Li et al., 2023; Liu et al., 2023). It enables the spatial localization and analysis of molecules at the single-cell level, providing a powerful detection method for revealing cellular heterogeneity and its mechanisms in disease initiation and progression (Siheun Lee et al., 2023; Li et al., 2023). Mass Spectrometry Imaging (MSI) is a label-free technique that offers detailed insights into the spatial distribution and abundance of target molecules in organisms (Zhang et al., 2024). Renowned for its sensitivity and specificity (Fletcher and Vickerman, 2013; Shon et al., 2014) MSI excels in analyzing endogenous metabolites in tissues and cells, making it valuable in medicine (Nilsson et al., 2015), life sciences, and pharmacy (Xie et al., 2024; Schulz et al., 2019; Philipp et al., 2024; Fletcher and Vickerman, 2013).

ToF-SIMS, a highly spatially resolved MSI technique, has been extensively employed in single-cell imaging analysis (Feifei et al., 2023). For instance, Passarelli et al. employed ToF-SIMS for single-cell metabolomic profiling to investigate the distribution of the drug amiodarone in macrophage cells (Rabia et al., 2020). Their study revealed that the upregulation of phospholipid species and cholesterol is associated with the accumulation of amiodarone. Pareek et al. employed metabolomics in conjunction with ToF-SIMS imaging to directly observe *de novo* purine biosynthesis mediated by the purinosome (Pareek et al., 2020). Similarly, Sparvero et al. utilized ToF-SIMS imaging to delineate the distribution of peroxidized phosphatidylethanolamine at the single-cell and subcellular levels, revealing its accumulation in pathological states associated with traumatic brain injury (Tian et al., 2017).

However, since ToF-SIMS is a technique based on high vacuum, sample preparation is a key factor for ToF-SIMS imaging analysis, especially how to better maintain the biological activity of cells for vacuum detection (Liu and Ouyang, 2013; Lee et al., 2012). Common sample preparation methods in SIMS include chemical fixation, freeze-fraction, and freeze-drying (Wu et al., 2017; Lanni et al., 2012). Chemical fixation refers to the use of chemical agents to alter the chemical properties of cells to maintain cell shape, avoiding to disrupt the integrity of the cell membrane and the distribution of molecules on the surface (Li et al., 2021; Braidy et al., 2014; Malm et al., 2009). Freeze-fraction involves sandwiching the cell suspension between two substrates and cryopreserving it until a moderate force is applied to fracture the two ice substrates and expose the cells (Berman et al., 2010; Colliver et al., 1997; Jia et al., 2023). The most common method of sample preparation used in current ToF-SIMS research is freeze-drying due to the convenience and effectiveness, in which the sample is rapidly frozen to preserve its chemical composition and then slowly dried under vacuum to remove any remaining water (Yoon and Lee, 2018; Jia et al., 2023). However, a significant challenge associated with this technique is the transfer of the sample from liquid nitrogen to the freeze-drying instrument. During this transfer, the low temperature of the sample surface can lead to the condensation of airborne contaminants, which may introduce extraneous signals and subsequently reduce the reproducibility of the results. One approach to ensuring a solvent-free environment is the utilization of a glove box filled with clean, dry nitrogen or maintained under vacuum conditions (Li et al., 2021; Barreto et al., 2014; Malm et al., 2009). However, glove boxes are costly and not commonly found in most analytical laboratories. Furthermore, there is a scarcity of detailed descriptions regarding lyophilization for sample preparation within the research literature (Bjarnholt et al., 2014), and a Standard Operating Procedure (SOP) is notably absent. Consequently, efforts to develop a sample preparation protocol should prioritize ease of use while simultaneously addressing potential sources of contamination.

In this study, we systematically optimized each stage of the cell sample processing procedure and provided comprehensive protocols. To assess the impact of these optimizations on the outcomes, we conducted a comparative analysis of single-cell imaging data obtained before and after the optimization process. Additionally, we compared the results of optimized freeze-drying sample preparation with those of chemical fixation to assist researchers in selecting the most suitable method based on their specific objectives. We expect that the detailed sample preparation processes we have provided can further advance the field of single-cell mass spectrometry imaging (MSI) research.

## 2 Materials and methods

### 2.1 Cell culture

Prior to cell seeding, the commercially polished silicon wafers were sectioned into approximately 1 cm × 1 cm squares using a diamond knife. Subsequently, the wafers underwent a series of cleaning procedures, which included ultrasonication in methanol, acetone, and deionized water for 10 min each. After cleaning, the wafers were allowed to air dry at room temperature. The cleaned wafers were then stored in sealed containers until required for use.

Huh-7 cells, obtained from the Cell Resource Center at the Institute of Basic Medical Sciences, CAMS/PUMC, China, were cultured in a high-glucose DMEM sourced from Gibco (C11995500BT). The medium was supplemented with 10% fetal bovine serum (FBS) from Sigma (F8318) and 1% penicillin-streptomycin from Gibco (15140122). The cells were maintained at 37°C in an atmosphere containing 5% CO_2_.

Once the cell culture attained approximately 80% confluence, the cells underwent two washes with PBS (Invitrogen, C10010500BT) and were subsequently exposed to 0.25% trypsin (Invitrogen, 25200072) for a duration of 2 min. This was followed by the addition of culture medium to halt the enzymatic digestion. The cells were then resuspended by gentle pipetting and seeded at a density of 1 × 10^4^ cells/cm^2^onto a culture dish containing Si wafers. They were incubated overnight for 12 h to facilitate adherence to the Si wafers.

### 2.2 Chemical fixation

The silicon wafers, which contained the seeded cells, were removed from the medium and underwent sequential washes in PBS and a 0.15 M ammonium formate (AF, Biorigin, BN24368) solution for 3 s, repeated twice. This was followed by fixation with glutaraldehyde (Biorigin, BN25007) for 15 min. Subsequently, the wafers were rapidly rinsed with a 0.15 M AF solution, followed by a quick rinse in ultrapure water. Finally, the wafers were analyzed after being dried at room temperature.

### 2.3 Freezing fixation

The silicon wafers, which contained the seeded cells, were meticulously extracted from the medium and immersed in PBS for 2–3 s. This washing procedure was repeated twice to ensure thorough cleansing. Subsequently, the wafers were promptly transferred into a 0.15 M AF solution for approximately 30 s, followed by sequential rinsing in three separate tubes to ensure the removal of any residual salt ions from the PBS. Excess liquid was removed using absorbent paper. Subsequently, under a nitrogen atmosphere, the silicon wafer was manually immersed in isopentane coolant, pre-cooled with liquid nitrogen, to achieve rapid freezing. The wafer was then carefully removed and transferred to a pre-cooled container, maintained under nitrogen protection.

### 2.4 Freeze-drying

The sample was subjected to lyophilization at a temperature of −55°C and a pressure of 10^−3^ mbar for a duration of 12 h (Christ, Alpha 1-4 LSCbasic). Following this, the sample was incrementally warmed to ambient temperature to promote the evaporation of any residual isopentane reagent. The desiccated sample is then suitable for analysis by ToF-SIMS.

### 2.5 ToF-SIMS data acquisition

ToF-SIMS measurements were carried out on a ToF-SIMS V instrument (ION-TOF GmbH, Münster, Germany). The cells were analyzed in an analysis-sputtering mode, employing a 30 KeV Bi_3_^+^ primary ion beam for the generation of mass spectrometry images, and a 10 KeV Ar_1600_^+^ sputtering beam to eliminate any contamination or chemical damage induced by the Bi_3_^+^ beam on the sample surface. In a spatial resolution of approximately 200 nm, the beam current for Bi_3_^+^ ions is 0.18 pA and for Ar_1600_^+^ ions is 9.0 nA. The sputtering area was 500 × 500 μm^2^, with the Bi_3_^+^ analysis beam scanned 350 × 350 μm^2^ with 256 × 256 pixels within the central area of the sputtering region (Winograd, 2018). Non-interlaced mode was applied and the parameters are as follows: analysis with 10 scans, sputtering with 1 scan, and a pause of 0.8 s. This cycle was iteratively repeated until the complete removal of all cells by sputtering. The accumulated data were subsequently compiled and presented as a final image. The spatial resolution of potassium ions in positive ion mode and phosphate ions in negative ion mode is approximately 200 nm and 500 nm, respectively. To mitigate the charge accumulation on the sample surface, a low-energy electron gun is employed.

In high mass resolution (HMR) mode, both positive and negative mass spectra were acquired. The beam current was approximately 0.8 pA with a pulse width of less than 1 ns, operating in Bunched Mode. The mass spectral resolution typically exceeded 5,000 for C_2_H^−^ (full width at half maximum, FWHM). The total ion current for each scan ranged from 10^11^ to 10^12^ ions/cm^2^, and the data were rasterized over a 256 × 256 pixel within a 300 × 300 μm^2^ area. The cycle time was 100 μs, corresponding to a mass spectrum range of 0–908 Da, with a frequency of approximately 10 kHz.

### 2.6 Data analysis

The mass spectra were processed using IONTOF SurfaceLab 7.0. The peak calibration was performed by C^−^、O^−^、OH^−^、C_2_H^−^、PO_3_^−^ and C_16_H_31_O_2_^−^ in negative ion mode, and C^+^, CH^+^, CH_3_^+^, C_2_H_3_^+^, C_3_H_5_^+^, C_5_H_12_N^+^ and C_5_H_15_PNO_4_^+^ in positive ion mode. A selection of mass spectrum peaks with signal exceeding 3,000 counts and SNR greater than 3.0 and the mass range is 0–900 Da.

Regions of interest (ROIs) were identified by applying a threshold based on the phospholipid fragment at m/z 184 in the positive ion mode and the phosphate group at m/z 79 in the negative ion mode. The relative signal intensity of individual cells was quantified by normalizing the total ion count across all pixels within each cell. Statistical processing and analysis of the data obtained from single-cell mass spectrometry were performed using R version 4.3.2 and Origin 2022.

## 3 Results and discussion

### 3.1 Protocol of freeze-drying sample preparation

**FIGURE 1:**
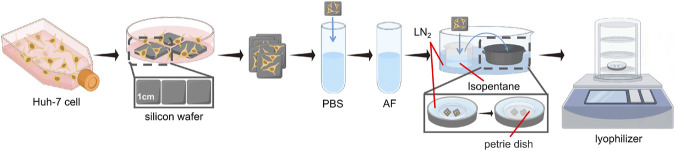
Optimised workflow for freeze-drying sample preparation of cell samples. LN_2_ is liquid nitrogen.

Huh-7 cells cultured on Si substrates were employed as a model to optimize the freeze-drying sample preparation. By the experience from prior studies (Zhang et al., 2022; Suvannapruk et al., 2022; Winograd and Bloom, 2015; Fletcher et al., 2011; Malm, 2009) and our own observations, we formulated a protocol designed to reduce molecule diffusion and surface contamination during freeze-drying as illustrated in Figure 1. Similar with other study, prior to freezing, it is essential to eliminate the medium and supplementary components from the cell surface by employing PBS solution washing. Following this, the PBS salts were rinsed off with 0.15 M volatile AF under isoosmotic conditions for 6–8 s, repeated 3 times to mitigate the effects of membrane salt on ToF-SIMS analysis. It is important to note that this procedure is cautious due to limited washing time, incomplete salt removal during rinsing, and the potential risk of excessive cell swelling and rupture with prolonged wash periods (Malm et al., 2009).

In the Freeze-drying process, high-purity isopentane was pre-cooled in liquid nitrogen at a temperature of −196°C until the liquid underwent a phase change, transitioning from clear to cloudy, signifying its attainment of a freezing point of −160°C, deemed ideal for cell preservation. Following this, the cell sample on the silicon wafer is immediately submerged in the pre-cooled isopentane coolant for rapid freezing. Isopentane, employed as a refrigerant, presents various advantages including accelerated cooling, improved thermal conductivity, a higher boiling point compared to conventional refrigerants such as propane, spontaneous evaporation at ambient temperatures, and enhanced safety protocols in experimental settings, thereby enabling rapid freezing and minimizing risks. Following the aforementioned treatment, the cell sample should be transferred to a Freeze-dryer machine. In light of the difficulties related to transporting frozen samples without a glove box in a cold, sterile environment, our choice was a stainless steel insulated bucket (or a foam box) with a cylindrical lid. This container provides ample space for manually handling the entire freezing process, including immersing the samples in isopentane for rapid freezing and quickly removing them, as well as placing a stainless steel tray containing the petri dish. Prior to freezing, isopentane, tweezers, the tray, and the petri dish (including its lid) should all be pre-cooled. In this procedure, there are no stringent size specifications for the stainless steel insulated bucket, foam box, or stainless steel tray; the primary consideration is their height. The insulated bucket or foam box must be sufficiently tall to ensure a specific distance between the top and the liquid nitrogen volume inside. Furthermore, the height of the stainless steel tray should exceed that of the petri dish to accommodate a small quantity of liquid nitrogen. This ensures that the petri dish remains uncontaminated for the placement of frozen silicon wafers, with the lid of the petri dish being closed once all wafers have been removed. When liquid nitrogen is exposed to warm air, it rapidly undergoes a phase transition from liquid to gas, forming a low-density nitrogen layer that covers both the interior and the surface of the insulated bucket or foam box. As an inert gas, nitrogen is non-reactive, which inhibits bacterial growth and protects the enclosed environment from oxygen and other oxidizing agents, thereby creating a controlled experimental environment under low temperature and minimal contamination. Furthermore, the nitrogen layer helps maintain a low sub-zero temperature, ensuring that no liquefaction occurs during the transfer of the samples from isopentane to the petri dish.

Subsequently, under nitrogen protection, the stainless steel tray containing the samples is transferred to the pre-cooled freeze-dryer, preparing for the freeze-drying process. During the transfer and while the freeze-dryer re-establishes the specified pressure, small particles or impurities in the low-vacuum air may condense or adhere to the sample surface. The liquid nitrogen inside the tray and the petri dish lid ensure that the transfer and drying process are completed at low temperatures and with minimal contamination risk. The liquid nitrogen level in the tray should be minimal, and the efficient operation of the vacuum pump ensures the complete vaporization of the liquid nitrogen inside the stainless steel container, without directly participating in the vacuum process.

### 3.2 Cell imaging after optimizing freeze-drying sample preparation

**FIGURE 2:**
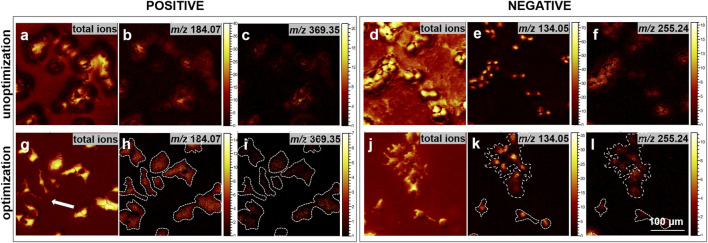
Comparison of ion images before and after the optimization of cell sample preparation. **(a–c, g–i)** depict ToF-SIMS ion images acquired in the positive ion mode. Specifically, **(a, g)** illustrate the total ion images derived from the initial 10 scans of cell imaging; **(b, h)** highlight the *m/z* 184.07, corresponding to the phosphocholine head group; **(c, i)** represent the *m/z* 369.35, associated with [cholesterol-OH]^+^. Conversely, **(d–f, j–l)** present ToF-SIMS images in the negative ion mode. **(d, j)** exhibit the total ion images from the first 10 scans of cell imaging; **(e, k)** focus on the *m/z* 134.05, serving as a nuclear marker; and **(f, l)** depict the *m/z* 255.24, indicative of FA16:0. Each image was acquired at 300 × 300 μm^2^ with a resolution of 256 × 256 pixels. Scale bar: 100 μm.

To assess the effectiveness of our optimized freeze-drying sample preparation protocol in minimizing the redistribution of intracellular small molecules and surface contamination, high spatial resolution ToF-SIMS was utilized for imaging the cell samples. It is noteworthy that the primary distinction between the unoptimized and optimized sample preparation lies in whether the process is conducted under the protection of liquid nitrogen. As depicted in Figure 2, the ToF-SIMS images presented the total ion signal from the initial ten scans in both positive and negative ion modes, along with the distribution of intensities of representative ion signals. In positive ion mode, *m/z* 184.10, typically associated with the head group fragment of phosphatidylcholine, and *m/z* 369.35, linked to cholesterol, were selected to evaluate the integrity of the cell membrane and cell morphology. Furthermore, the distributions of *m/z* 255.24, attributed to FA 16:0, and *m/z* 134.05, which may correspond to adenine (Ade-H) and serve as a nuclear marker fragment for cell nucleus localization, were analyzed in negative ion mode. The results suggest that compared to directly placing the samples in the freeze-dryer without nitrogen protection, the addition of cryoprotectants and the nitrogen protection during the transfer and drying process (the optimized transfer and drying process) significantly reduces contamination on the cell surface and background. Furthermore, the specific ion images reveal more distinct cell boundaries, as highlighted by the white arrow, demonstrating improved cell morphology integrity and reduced cell rupture. Additionally, molecular diffusion is more pronounced prior to optimization, leading to an enhanced movement and dispersion of molecules from the interior cells to the cell boundary, which consequently causes the boundary to appear blurred. These findings indicate that the optimized transfer-drying process significantly minimizes contamination on both the cell surface and the background.

**FIGURE 3:**
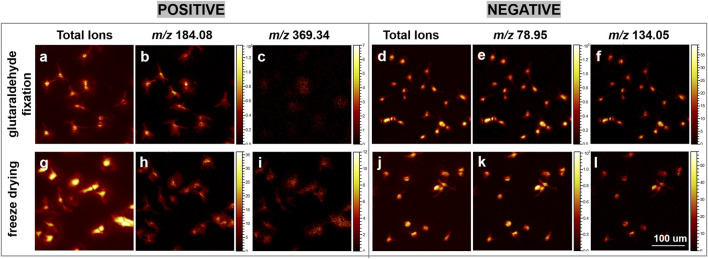
A comparative analysis of ion images obtained from glutaraldehyde-fixed and freeze-dried cells using gas cluster ion beam (GCIB) sputtering mode. **(a–c, g–i)** present ToF-SIMS ion images captured in the positive ion mode. Specifically, **(a, g)** illustrate the total ion images derived from cellular imaging; **(b, h)** depict the ion at *m/z* 184.08, corresponding to the phosphocholine head group; and **(c, i)** represent the ion at *m/z* 369.34, associated with [cholesterol-OH]^+^. **(d–f, j–l)** exhibit ToF-SIMS ion images in the negative ion mode. **(d, j)** show the total ion images from cellular imaging; **(e, k)** display the ion at *m/z* 78.95, indicative of PO_3_^+^; and **(f, l)** represent the ion at *m/z* 134.05, a nuclear marker. Each image was acquired over an area of 300 × 300 μm^2^ with a resolution of 256 × 256 pixels. Scale bar: 100 μm.

Glutaraldehyde chemical fixation is a highly effective technique for preserving cellular integrity and morphology. This is attributed to glutaraldehyde’s role as a fixative, which primarily crosslinks amino groups in phosphatidylserine and phosphatidylethanolamine (Schelkle et al., 2017; Nagata et al., 2014), key constituents of the inner leaflet of the cell membrane, thereby maintaining cellular structural integrity (Zhang et al., 2022). To investigate the impact of freeze-drying sample preparation methods on cellular samples, we conducted a comparative analysis of glutaraldehyde chemical fixation and freeze-drying techniques. The influence of diverse sample preparation methods on cell morphology can be effectively demonstrated through the application of microscopy and mass spectrometry imaging to investigate distinct cellular regions. As depicted in Figure 3, although the filopodial signals are more pronounced under glutaraldehyde fixation, the optimized freeze-drying method also preserves a clear and intact cellular membrane structure comparable to that achieved with glutaraldehyde fixation.

**FIGURE 4:**
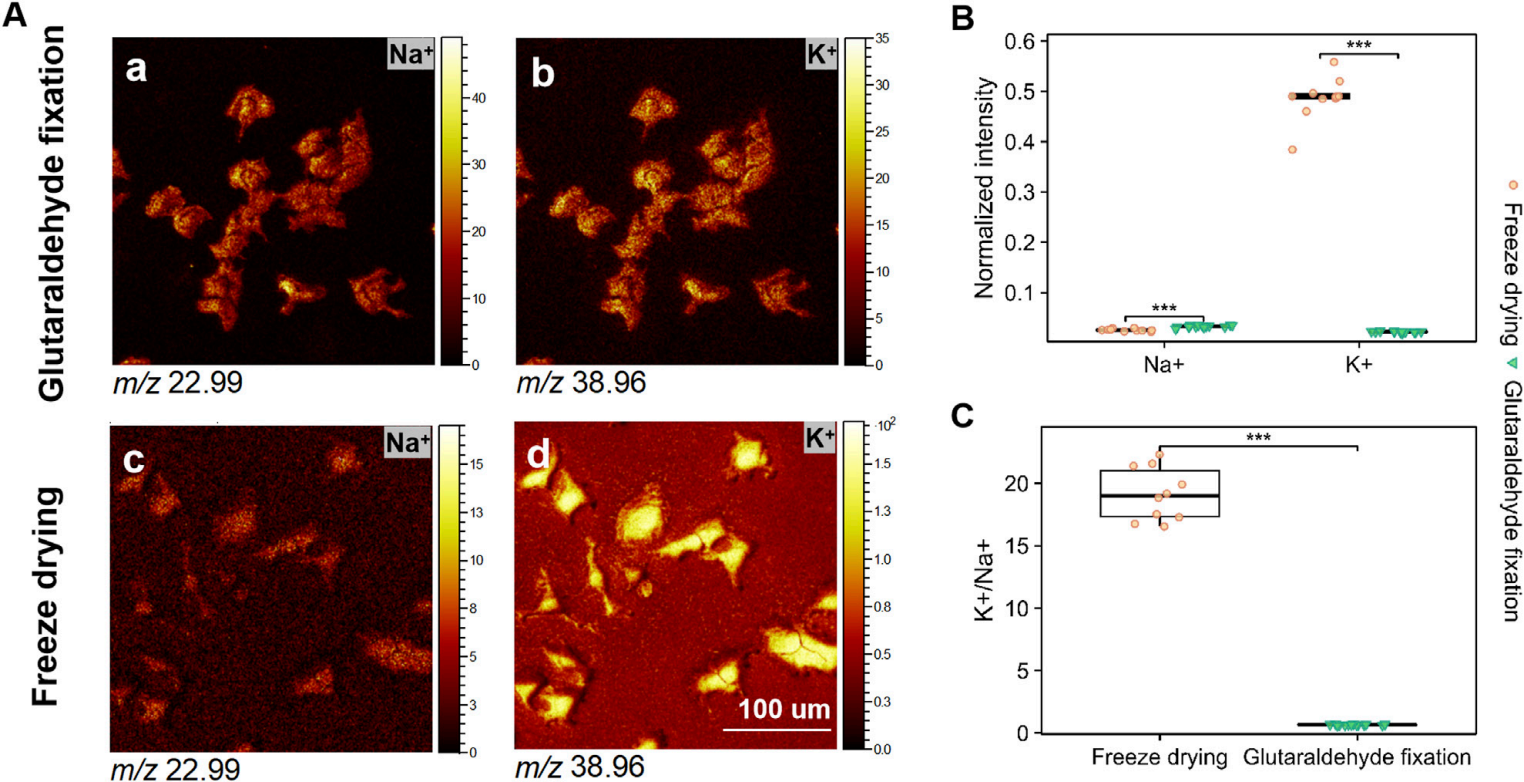
The imaging of K^+^ and Na^+^ signals, along with their ratios on glutaraldehyde fixation and freeze-drying samples using GCIB sputtering in positive ion mode. **(A)** Presents the imaging results for *m/z* 22.99 (Na^+^) and *m/z* 38.96 (K^+^) during the initial 10 scans. **(B)** Depicts the ion abundance of Na^+^ and K^+^ detected across the entire cell under sputtering conditions. **(C)** Provides a box plot representing the K^+^/Na^+^ ratio, highlighting the median, quartiles, and outliers. All images were acquired over a 300 × 300 μm^2^ area with a resolution of 256 × 256 pixels. The scale bar represents 100 μm.

Prior research suggests (Malm et al., 2009) that the potassium-to-sodium (K/Na) ratio serves as a critical parameter for assessing the efficacy of sample preparation techniques. The difference between glutaraldehyde fixation and freeze-drying is that the latter method enables the rapid preservation of cells in a viable state, thereby optimizing the retention of intracellular substance abundance (Gulin et al., 2020; Malm, 2009). Figure 4A depicts the distribution of K^+^ and Na^+^ signal intensities from samples treated with glutaraldehyde fixation and freeze-drying. Figure 4A depicts the distribution of K^+^ and Na^+^ signal intensities across samples that underwent glutaraldehyde fixation and freeze-drying processes. The data clearly demonstrate that freeze-drying cells retained a higher Concentration of K^+^ (Figure 4B), whereas the K^+^ signal was markedly diminished in cells subjected to glutaraldehyde fixation. This observation aligns with the findings of Grovenor et al. (2006), who assert that the sodium/potassium gradient in viable cells is maintained by active ion pumps and channels. Furthermore, rapid freezing at low temperatures effectively preserves elevated K^+^ concentrations while minimizing intracellular diffusion.

To achieve the optimal K/Na ratio, we conducted sputter analyses on each cell group until cellular signals vanished. The data reveal that the Na^+^ intensity is significantly reduced in freeze-dried cells compared to those fixed with glutaraldehyde, exhibiting approximately a two-fold decrease. Conversely, the K^+^ intensity is substantially elevated in freeze-dried cells relative to glutaraldehyde-fixed cells, where it is lower than the Na^+^ intensity. The K/Na ratio in freeze-dried samples is approximately 20, whereas it is less than 1 in samples prepared with glutaraldehyde fixation (Figure 4C). This underscores the importance of precise temperature and time control in freeze-drying for preserving intracellular substances. Although glutaraldehyde fixation enhances membrane clarity, it results in a lower K^+^/Na^+^ ratio, indicating less effective retention of intracellular substances. This phenomenon may be attributed to the action of glutaraldehyde as a fixative, which crosslinks amino groups in essential membrane components (Schelkle et al., 2017; Nagata et al., 2014). While this process preserves cellular structure, it concurrently disrupts the sodium-potassium balance (Zhang et al., 2022).

### 3.3 Comparison of molecular information under the treatment of different sample preparation

**FIGURE 5:**
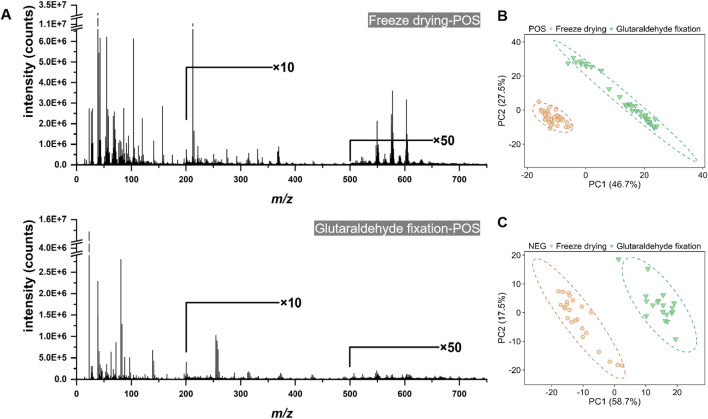
The ToF-SIMS positive ion mass spectra and principal component analysis (PCA) score plots of cell samples subjected to freeze-drying and glutaraldehyde fixation. **(A)** The spectra of both the freeze-drying and glutaraldehyde fixation groups, demonstrating the full mass range under consistent technical parameters. **(B)** The PCA score plot in the positive ion mode. **(C)** The PCA score plot in the negative ion mode.

In addition to preserving cellular morphology, the molecular information derived from cell samples is of paramount importance. Consequently, we employed ToF-SIMS in high mass resolution (HMR) mode to evaluate the effects of chemical composition under various sample preparation methods, including glutaraldehyde fixation and freeze-drying. We acquired signals from the cell membrane and subsequently performed background subtraction of signals from the cell population to obtain mass spectrometry data for the region of interest (ROI). This was designed to minimize instrumental errors arising from fluctuations in raw ion counts and sample topography. Figure 5 and Supplementary Figure S1 present the representative mass spectra for various sample preparations in both positive and negative ion modes. The mass spectra from different samples revealed numerous peaks with varying signal intensities, irrespective of cell type. In the positive ion mode, the low mass region (m/z < 150) of the mass spectra for cells prepared using different methods exhibited multiple fragment ion peaks associated with salt ions (Na^+^, K^+^), amino acids, lipids, and other organic molecules. A pronounced peak corresponding to phosphocholine was detected at *m/z* 184, accompanied by two distinct peaks attributable to cholesterol at *m/z* 369 and *m/z* 385 (refer to Figure 5). In the negative ion mode, the *m/z* 100–400 range predominantly exhibited fragment ions of fatty acids (Supplementary Figure S1B). In the higher mass range (*m/z* 500–760) under positive ionization mode, particularly for peaks likely indicative of diacylglycerol (DAG) or phosphatidylcholine molecular ions, as well as in the *m/z* 300–400 range, predominantly comprising molecular ion fragments of long-chain fatty acids under negative ionization mode, the mass spectral signal intensity was significantly enhanced in freeze-dried samples.

During the data collection process, spectra are obtained from the cellular region. Following the subtraction of background noise from ROI, the relative abundances of substances become comparable. Subsequently, pixel averaging is performed, and the relative intensity is determined by normalizing the total ion count. Principal component analysis (PCA) is then employed to compare two differently treated cell samples. Figures 5B, C illustrate that the identical cell population displayed distinct clustering patterns in the PCA score plots, with PC1 accounting for 46.7% and 58.7% of the variance, respectively. The majority of data points were located in the negative regions of PC1 and PC2, particularly in the positive ion mode. In the score plot, a separation between the two groups is evident along the PC1 axis (Supplementary Figure S2A). The corresponding loadings plot for PC1 reveals the *m/z* values responsible for the observed differences between the two cell groups in both positive and negative ion modes (Supplementary Figures S2B, C). Through analysis of the corresponding PC1 loading plot, a series of peaks were observed that are potentially associated with lipids, including *m/z* 369 (+), *m/z* 385 (−), *m/z* 255 (−), *m/z* 279 (−), *m/z* 281 (−), *m/z* 86 (+), *m/z* 125 (+), *m/z* 184 (+), and *m/z* 224 (+). These findings suggest that the abundance of these lipid-related substances is elevated during the freeze-drying process as opposed to glutaraldehyde fixation, indicating a variation in chemical composition between the two treatment groups.

To directly evaluate the alterations in the abundance of various chemical constituents, we have conducted a clustering analysis utilizing the selected adduct ion peaks. This was complemented by heatmap visualization to depict the relative intensity variations of different substances within the cells. Supplementary Figure S3A presents the normalized intensity values of a substantial number of cells across different groups, demonstrating strong reproducibility within each treatment group and a significant degree of differentiation between the groups. In the analysis of negative ions, the abundance of ions within the *m/z* 200–500 range was generally greater in freeze-dried samples compared to those fixed with glutaraldehyde. The ion peaks observed in this range primarily correspond to FAs. Conversely, in the positive ion mode, the abundance of ad ions in the *m/z* 0–300 range was lower in the freeze-dried samples than in the glutaraldehyde-fixed group. We systematically summarized the relevant peaks associated with cholesterol, FAs, and PC by examining their normalized relative intensities (Supplementary Figure S3B). To evaluate the statistical significance of lipid alterations between the two groups, we employed a *t*-test. The findings corroborated the results from the previously discussed PCA loading plot, consistently indicating elevated lipid content.

**FIGURE 6:**
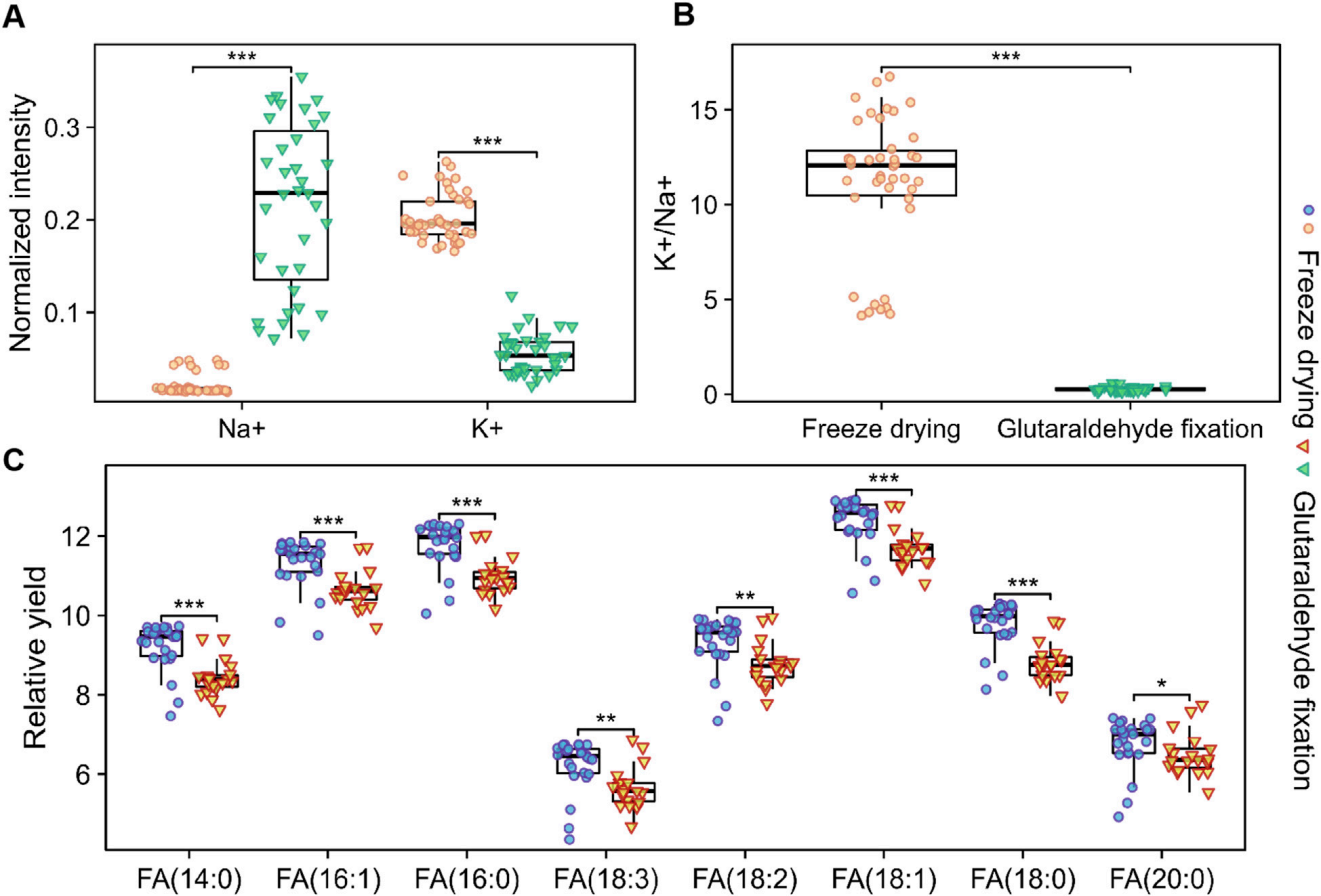
Box plots of the abundance and relative yields of Na^+^, K^+^, and various fatty acids in cells prepared using different sample preparation methods. **(A)** Relative abundance and ratios **(B)** of Na^+^ and K^+^ in the positive ion mode. **(C)** Relative yields of different fatty acids in the negative ion mode. Under stable ion dose conditions, the grouped box plots display the maximum, minimum, and median yields of each substance. Welch’s *t*-test was used to assess the differences between cells prepared by different methods, with p values <0.001 indicated by “***”.

It is crucial to acknowledge that the mass spectral signals indicative of cell membrane integrity revealed substantial variations in the peak signals of various substances. These alterations in signal intensity and relative abundance imply that they cannot be solely attributed to discrepancies in sample preparation techniques, such as the chemical cross-linking mechanisms inherent in glutaraldehyde fixation. Instead, they may also be associated with differences in secondary ion yield. Glutaraldehyde, when employed as a fixative, stabilizes cellular structures via chemical cross-linking, which can induce structural alterations or degradation of specific lipids. Variations in chemical composition and matrix effects arising from diverse sample preparation methods may increase sensitivity to critical lipid molecules, consequently influencing the relative ion yield from various components within the analyte. Under conditions of consistent ion dosage, we performed a comparative yield analysis of the components within the cell membranes across different sample treatment groups. Figures 6A, B present our investigations into the distribution of Na^+^ and K^+^ at the cellular membrane level, serving as an assessment of sample preparation. The findings corroborate the conclusions derived from sputtering, demonstrating an enhanced retention of K^+^. Although glutaraldehyde aids in the stabilization of pyridine derivatives for fixation purposes, it concurrently alters the sodium-potassium balance within the cell membrane.

**FIGURE 7:**
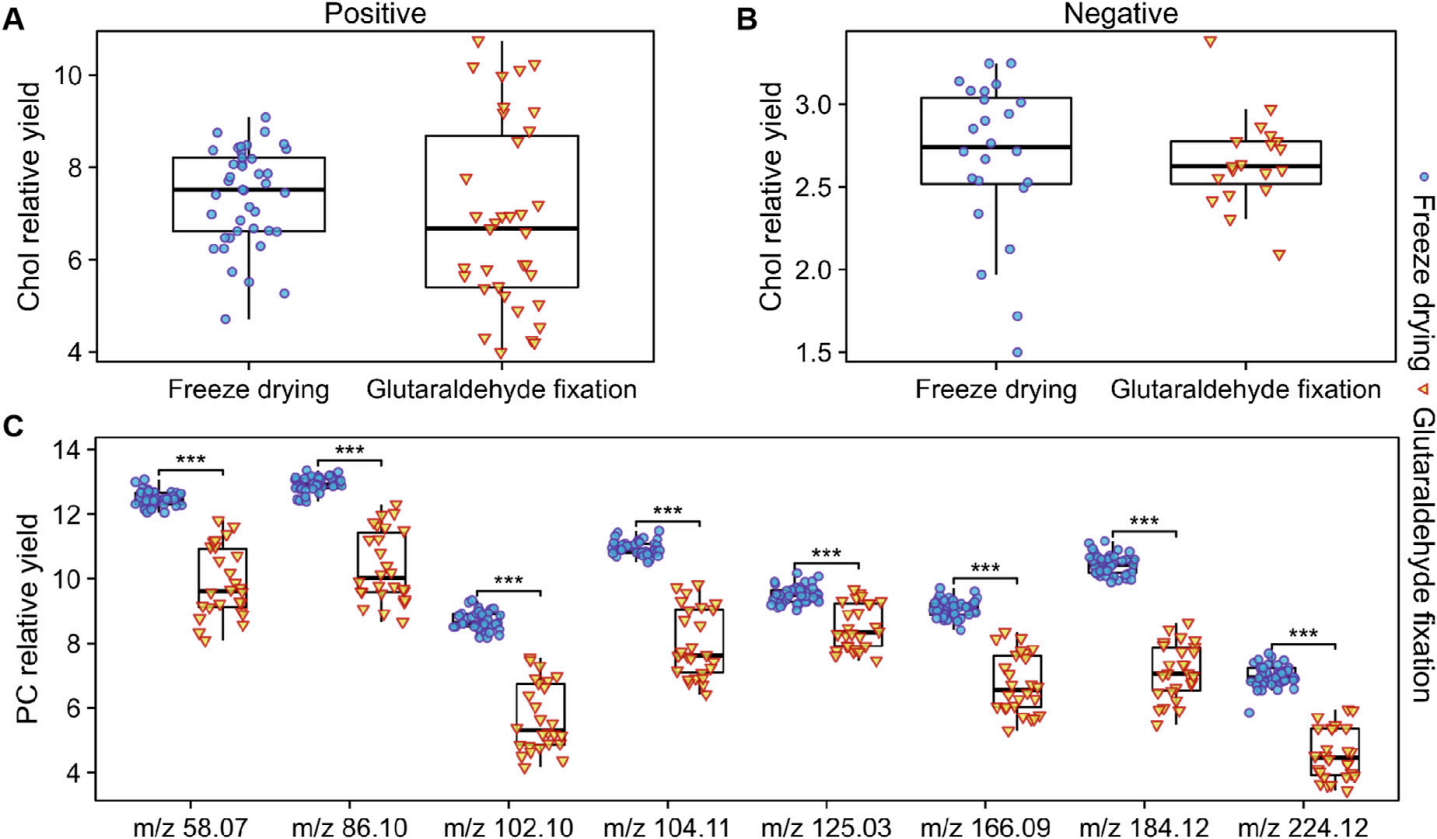
Box plots of the relative yields of cholesterol and PC-related peaks obtained under different sample preparation methods for various cells. **(A)** Relative yields of cholesterol-related peaks in the positive ion mode. **(B)** Relative yields of cholesterol-related peaks in the negative ion mode. **(C)** Relative yields of PC-related peaks in the positive ion mode. Under stable ion dosage conditions, the grouped box plots display the maximum/minimum yields and medians for each substance. Welch’s *t*-test was used to assess the differences between cells prepared by different methods, with p values <0.001 indicated by “***”.

In the negative ion mode, we compared the relative yields of eight FA with good signal quality from two groups of cell samples. Figure 6C presents the average values and outliers of each group, revealing that the yields of the eight FAs detected in the freeze-dried group were consistently higher than those fixed with glutaraldehyde. In the positive ion mode, we compared the peaks related to PC fragments (Figure 7C) and found that the yields under freeze-drying were also higher than those under glutaraldehyde fixation. The yields of FAs and PCs among the different treatment groups showed significant differences. Interestingly, when analyzing cholesterol under different modes, no significant differences were observed between the two groups for *m/z* 369 in positive ion mode and *m/z* 385 in negative ion mode (Figures 7A, B) mode across different batches. However, in the analysis of normalized relative intensities (Supplementary Figures S2, S3), it was noted that the cholesterol content in the cell membranes was higher in the freeze-dried samples compared to those fixed with glutaraldehyde. As shown in Figures 3C, I, cholesterol exhibited better abundance and distribution retention in the whole-cell sputtering imaging. Glutaraldehyde crosslinks proteins within the cell membrane resulting in a stable structure. This crosslinking action enhances the rigidity and stability of the cell membrane, potentially affecting the dynamic distribution of membrane lipids (Bik et al., 2020; Hobro and Smith, 2017), including cholesterol. Furthermore, this effect may be influenced by the washing steps following glutaraldehyde fixation, which can remove loosely bound, low molecular weight, or lipophilic substances such as phospholipids, certain cholesterol molecules, and other lipids (Zhang et al., 2022). These compounds may be partially lost or transferred into the solution during washing, thereby altering the original state and potentially impacting subsequent analyses. In contrast, freeze-drying effectively preserves cell structure, reducing the likelihood of degradation or changes to original compounds during analysis, thus maintaining the stability and integrity of the analytes. These conclusions further indicate that the different sample preparations caused by glutaraldehyde fixation and freeze-drying lead to significant differences in chemical composition, ionization efficiency, and signal generation, which directly impact imaging quality and the relative quantitative analysis of lipid components. Nevertheless, ToF-SIMS under various sample preparations can still analyze single cells and reveal the distribution of important molecular species within them.

## 4 Conclusion

This study illustrates a systematic approach about freeze-drying cell sample preparation without the use of glovebox, utilizing the Huh-7 cell line as a model. The results indicate that the use of liquid nitrogen for protection during the transfer process effectively reduces contamination of the cell surface and background. Furthermore, the optimized freeze-drying protocol exhibits superior efficacy in maintaining the structural integrity of large molecular entities, such as macromolecular lipids and fatty acids, within cells, in comparison to glutaraldehyde fixation. While glutaraldehyde fixation is more effective in preserving the integrity of substances with smaller molecular masses, researchers should choose the appropriate protocol based on their specific experimental needs. Ultimately, the optimized freeze-drying protocol is particularly well-suited for high-resolution single-cell culture analysis at both the cellular and subcellular levels.

## Data Availability

The raw data supporting the conclusions of this article will be made available by the authors, without undue reservation.
