# Supplementary material for: A laboratory-friendly protocol for freeze-drying sample preparation in ToF-SIMS single-cell imaging
Source: Front Chem. 2025 Mar 7;13:1523712. doi: 10.3389/fchem.2025.1523712 (PMC11925917; doi:10.3389/fchem.2025.1523712)
Supplement: Supplementary file 1 [file DataSheet1.docx]

Supplementary Material

# Supplementary Figures

**
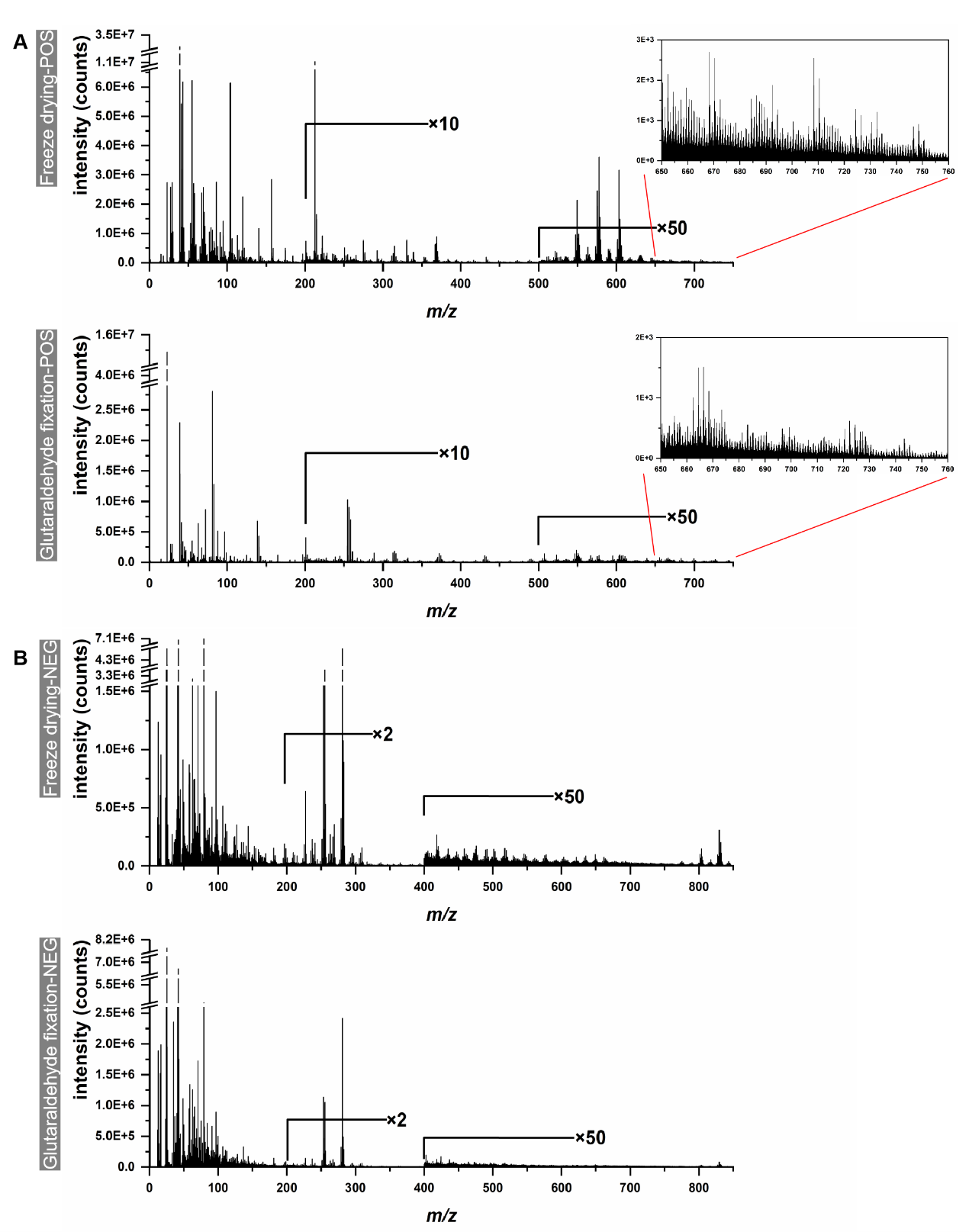
**

**Supplementary Figure 1.** Total ion mass spectra of different cell samples detected in the positive and negative ion modes, using freeze-drying and glutaraldehyde fixation, within the *m/z* range of 0-850. **A**. Mass spectrum in positive ion mode and the enlarged spectrum for the *m/z* range of 650-750. **B**. Mass spectra of different treatment groups in negative ion mode.

**
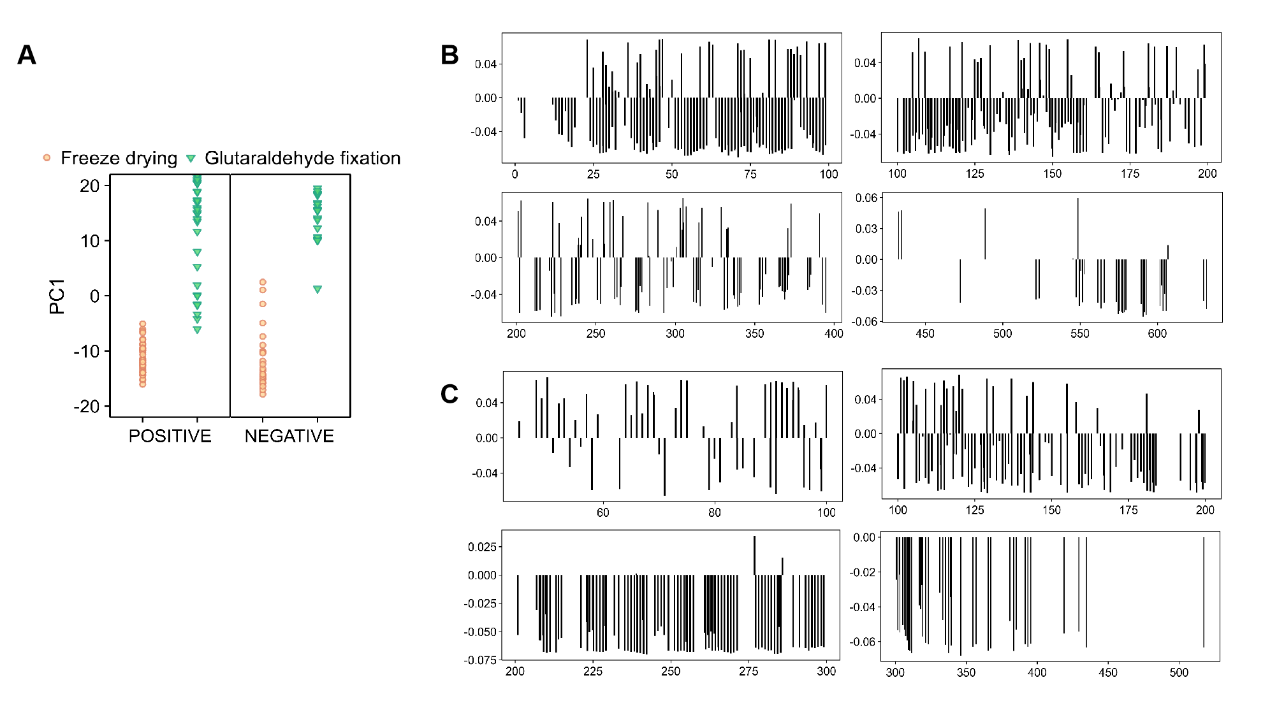
**

**Supplementary Figure 2.** PC1 score plot and corresponding loading plots for different treatment groups. **A**. PC1 score plot. **B**. Loading plot of PC1 in the mass range of 0-650 under positive ion detection. **C**. Loading plot of PC1 in the mass range of 0-520 under negative ion detection.


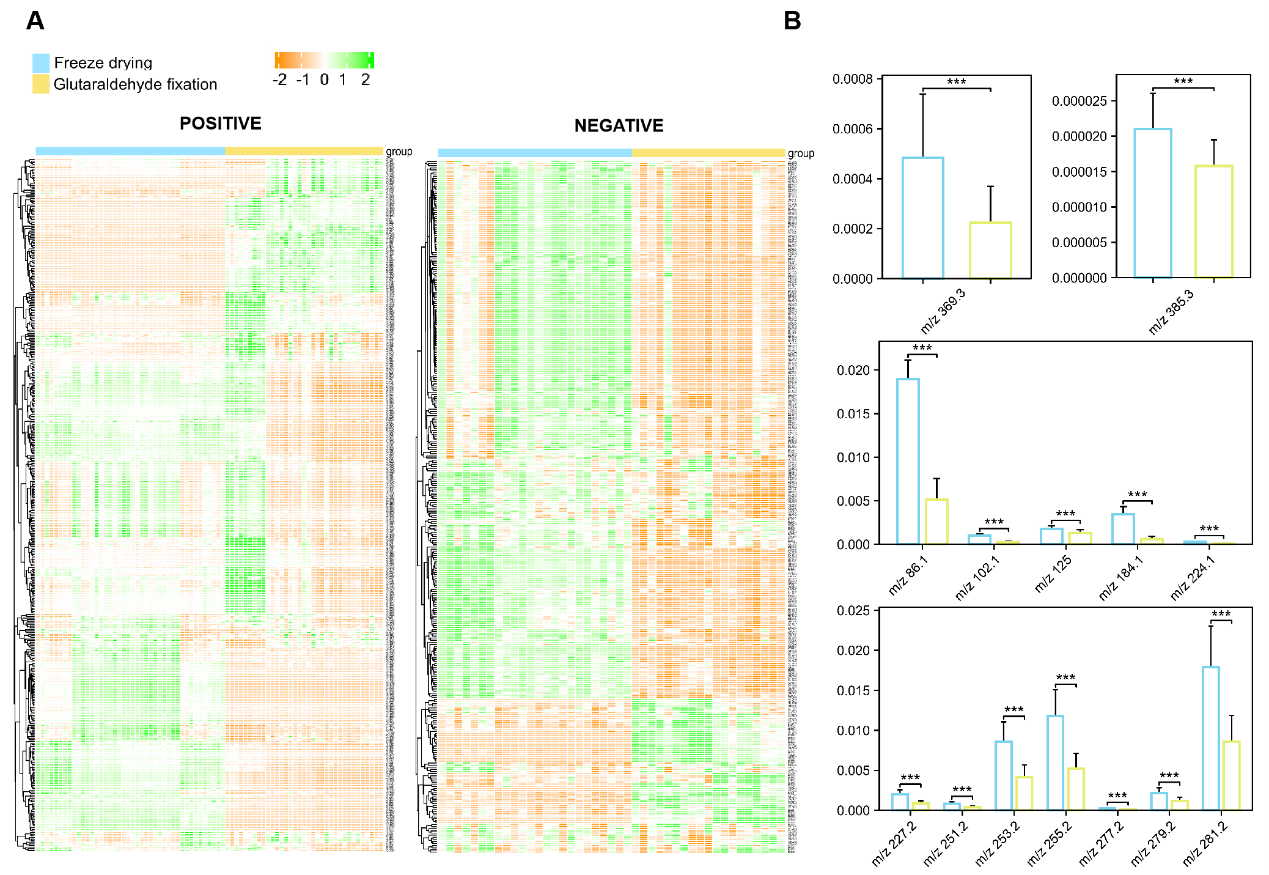


**Supplementary Figure 3.** Heatmap visualization of relative intensities of various substances across different sample groups in positive and negative ion modes, along with *t*-test comparisons. **A**. Heatmap analysis in positive and negative ion modes. **B**. Comparison of relative intensities of cholesterol, FAs, and PC related peaks in both ion modes.
